# Sexual dimorphism in the peripheral metabolic homeostasis and behavior in the TgF344‐AD rat model of Alzheimer's disease

**DOI:** 10.1111/acel.13854

**Published:** 2023-04-24

**Authors:** Hemant Srivastava, Alexander Tate Lasher, Akash Nagarajan, Liou Y. Sun

**Affiliations:** ^1^ Department of Biology University of Alabama at Birmingham Birmingham Alabama USA

**Keywords:** Aging, Alzheimer's disease, insulin, glucose, metabolism

## Abstract

Alzheimer's disease (AD), a prevalent form of dementia, is characterized by the decline of cognitive abilities with age. Available treatment options for AD are limited, making it a significant public health concern. Recent research suggests that metabolic dysfunction plays a role in the development of AD. In addition, insulin therapy has been shown to improve memory in patients with cognitive decline. In this study, we report the first examination of body composition, peripheral insulin sensitivity, and glucose tolerance in relation to behavioral assessments of learning, memory, and anxiety in the TgF344‐AD rat model of AD. Results from glucose and insulin tolerance tests show that female TgF344‐AD rats exhibit impaired glucose clearance and reduced insulin sensitivity at both 9 and 12 months of age, while males display no differences at 9 months and even improved glucose clearance at 12 months. Results from the Morris Water Maze assessment of learning and memory reveal that male TgF344‐AD rats display impairments at both 9 and 12 months of age, while female TgF344‐AD rats only show impairments at 12 months. Furthermore, results from open field and elevated plus maze tests suggest that female TgF344‐AD rats display increased anxiety at 9 months of age; however, no differences were detected in males or at 12 months of age. Overall, our findings suggest that impairments in metabolism, commonly associated with type 2 diabetes, occur before or simultaneously with cognitive decline and anxiety in a sexually dimorphic manner in the TgF344‐AD rat model.

## Graphical abstract

Impaired glucose clearance and reduced insulin sensitivity were observed in female TgF344‐AD rats, while males showed no differences. Male rats had learning and memory impairments at both 9 and 12 months of age, while females showed impairments only at 12 months. The findings suggest that metabolic issues occur simultaneously with cognitive decline and anxiety in a sexually dimorphic manner in the TgF344‐AD rat model.

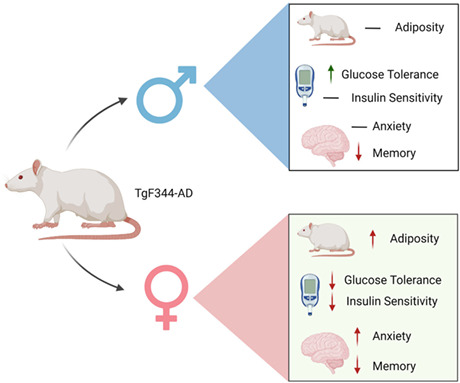

## 1 INTRODUCTION

Alzheimer's disease (AD) is a growing public health concern in both the United States and the world. For Americans aged 65 or older, AD represents the fifth leading cause of death (“2020 Alzheimer's disease facts and figures,” 2020), and it is estimated that over 80 million people worldwide will be living with some form of dementia by 2040 with AD comprising a majority of these (Ballard et al., 2011). AD is broadly characterized by an irreversible progressive loss of cognition and memory often accompanied by anxiety‐like behavior as well as the development of hallmark amyloid‐β accumulation, hyperphosphorylated‐Tau aggregation, and neuroinflammation within the brain (Donovan et al., 2018; Jack et al., 2018; Reitz et al., 2011). Despite a sizeable body of research targeting these hallmarks of AD, progress in developing effective treatments has been limited (Kumar et al., 2015; Mehta et al., 2017), necessitating further research into the features of this disease.

Epidemiological studies show that adult humans with type‐2 diabetes have a 50%–100% increased risk of developing dementia compared to those without (Biessels et al., 2006) and proteomic analyses of AD patient brains implicate carbohydrate metabolism among the differently regulated pathways associated with the disease (Johnson et al., 2020). In addition, postmortem analysis of brain tissues from AD patients shows a marked reduction in insulin‐like growth factor‐I (IGF‐I), IGF‐II, their respective receptors, and the insulin receptor at both the protein and mRNA levels (Steen et al., 2005). Targeting this pathway shows promise, as intravenous (IV) insulin administration to AD patients has improved recall memory independent of blood glucose levels (Craft et al., 1999) and intranasal insulin administration similarly improves recall memory in some AD patients while navigating the risk of hypoglycemia associated with IV insulin (Craft et al., 2017; Reger et al., 2006, 2008). While promising, these studies also suggest that insulin‐mediated improvements in AD patients likely depend on genetic background, as *APOE‐ε4* allele carriers did not benefit from treatment (Reger et al., 2006, 2008). In addition, the utilization of a long‐acting insulin analog did not improve memory in AD patients when compared to placebo treatment while regular insulin did (Craft et al., 2017). These caveats indicate that a more complete understanding of the insulin/IGF signaling pathway dysregulation is necessary if this avenue of treatment is to be further pursued.

Rodent models of AD demonstrate the importance of metabolic deficits in the progression of the disease. Transgenic mice overexpressing the human *APP* gene display defects in peripheral insulin sensitivity before amyloid‐β accumulation in the brain and cognitive decline (Hendrickx et al., 2021). Double transgenic *APP/PS1* overexpressing mice show diminished glucose tolerance and insulin sensitivity before cognitive impairment that coincides with amyloid‐β plaque formation (Macklin et al., 2017), and 3xTg‐AD mice display disrupted central insulin signaling along with reduced glucose tolerance and elevated body weight in adulthood (Velazquez et al., 2017). It is generally thought that AD begins years before the onset of dementia (Price & Morris, 1999), thus characterizing the changes in peripheral metabolism that occur before the onset of behavioral changes is of particular importance as it may be an important component of AD progression. To this end, we employed the TgF344‐AD transgenic rat model which expresses both mutant human amyloid precursor protein (*APP*_*SW*_) and presenilin 1 *(PS1ΔE9)*, resulting in the recapitulation of age‐dependent AD pathology including elevated soluble amyloid‐β, hyperphosphorylated tau, neuronal loss, and gliosis as early as 6 months of age. These characteristics make the TgF344‐AD rat model an attractive pre‐clinical model of AD for the elucidation of early alterations in metabolism that accompany the disease, as transgenic mouse models generally lack one or more of these features (Cohen et al., 2013). In this study, we hypothesized that adipose accumulation and impaired glucose/insulin tolerance typically associated with pre‐diabetes would be detectable before the onset of the cognitive symptoms of AD. Here, we carried out what we believe to be the first assessment of body composition analysis, glucose tolerance testing, and insulin tolerance testing in both sexes. We carried out these metabolic tests in conjunction with behavioral assessment to characterize the development of mental decline and metabolic dysregulation in this model of AD.

## 2 MATERIALS AND METHODS

### 2.1 Animals

All procedures used in this experiment have been approved by the National Institute of Health Office of Animal Care and Use and followed the guidelines set by the Institutional Animal Care and Use Committee of the University of Alabama at Birmingham.

A pair of TgF344‐AD^+^ breeders (wild‐type females and transgenic males) were obtained from Dr. Terrence Town of University of Southern California, and these were bred to produce transgenic (Tg) and wild‐type (WT) male and female offspring. All offspring were weaned at 21 days of age and tail biopsies were genotyped as previously described (Cohen et al., 2013). The rats were separated by sex and group housed at a density of 1–4 rats per cage in a facility that maintained a normal 12‐h light/dark cycle with ad libitum access to standard rodent chow and water. Different cohorts of animals were used for all tests conducted at 9‐month old and 12‐month old in an effort to reduce the impacts from repeated testing. Prior to all behavior testing, animals were habituated to handling for 14 days and acclimated to the testing room for 1 h in their home cages before being handled.

### 2.2 Quantitative magnetic resonance

Body composition was determined using the EchoMRI Whole Body Composition Analyzer for rats in conjunction with EchoMRI 2018 Body Composition Analyzer software. Unanesthetized rats were placed into the Body Composition Analyzer holding tube in a prostrate position and scanned. Data obtained included lean mass and fat mass. Body composition measurements were conducted by the University of Alabama at Birmingham Small Animal Phenotyping Core.

### 2.3 Glucose and insulin tolerance tests

For glucose tolerance testing, animals were fasted overnight (approximately 16 h) and injected intraperitoneally with 1 g/kg glucose prepared as 40% w/v in 0.9% saline solution. For insulin tolerance testing, animals were fasted 4 h and injected intraperitoneally with 7 IU/kg porcine insulin (Sigma‐Aldrich) prepared as 0.03 IU/μL in 0.9% saline solution. Tail vein blood glucose measurements were taken prior to injection (“minute 0”) and at time points indicated following injection using an AgaMatrix PRESTO blood glucometer. Blood glucose was compared between groups at each time point, and area under the curve (AUC) was analyzed to compare glycemic excursion for the duration of the test. For glucose tolerance test (GTT), incremental AUC was calculated where only values above the baseline glycemia we included to account for differences in basal glucose levels. For insulin tolerance test (ITT), net AUC was calculated where no baseline correction was made. All tests took place at the same time of day to avoid circadian rhythm bias.

### 2.4 Plasma collection and ELISA

Whole blood was collected from the tail vein into EDTA‐coated tubes from 12‐month‐old rats with ad‐lib access to standard rodent chow following isoflurane anesthesia. Plasma was collected by centrifugation at 2000 ×*g* for 10 min at 4°C. Plasma insulin concentration was determined using a Chrystal Chem ultrasensitive insulin ELISA kit (catalog #90080) according to the manufacturer's instructions.

### 2.5 Morris water maze

The Morris water maze test was used to assess spatial learning and long‐term memory over the span of 5 days. The apparatus used was a blue plastic pool that is 183 cm in diameter and was filled with 18–20°C water. A transparent platform 10 cm in diameter was placed 0.5 cm underneath the surface of the water in the northwestern quadrant of the pool. During the first 4 days, rats were subjected to four training trials each beginning from one of the four cardinal points (north, south, east, or west) of the pool. Each trial consisted of a rat freely searching for the platform for 2 min and concluding once the rat remained on the platform for 5 s. If a rat was unable to find the platform after 2 min, they were guided to the platform. On day 5, the average latency for each rat to find the platform was recorded. Following the four trials on this day, a probe trial was conducted where the platform was removed, and rats were allowed to freely swim for 60 s in the pool while being tracked by a motion camera at 15 frames/s (Noldus Ethovision 14).

### 2.6 Elevated plus maze

The elevated plus maze (EPM) was used to assess anxiety‐like behavior. The maze consisted of four 60 cm long, 10 cm wide arms located 60 cm above the ground. Two “closed arms” of the maze were surrounded by 60 cm high opaque walls while the other two “open arms” had no walls. Each animal was placed in the center of the maze and allowed to freely roam for 4 min while being tracked by a motion camera at 15 frames/s (Noldus Ethovision 14).

### 2.7 Open field test

The open field (OF) test was also used to assess anxiety‐like behavior. The OF arena consists of an opaque, square Plexiglass chamber measuring 90 cm × 90 cm with 40 cm high walls. Rats were placed in the same corner to begin each trial and were allowed to roam the field for a 4‐min period while being tracked by a motion camera at 15 frames/second (Noldus Ethovision 14).

### 2.8 Statistical analysis

Means of two groups were compared using the unpaired two‐tailed *t*‐test with the welch correction applied where appropriate. Where group means of different genotypes and sexes were compared, two‐way ANOVA was used with post‐hoc testing applied where significant differences were detected. The Benjamini and Hochberg correction was applied to post‐hoc tests. Means were considered significantly different at *p* < 0.05. Analyses were carried out and figures were generated using the R programming language.

## 3 RESULTS

**FIGURE 1:**
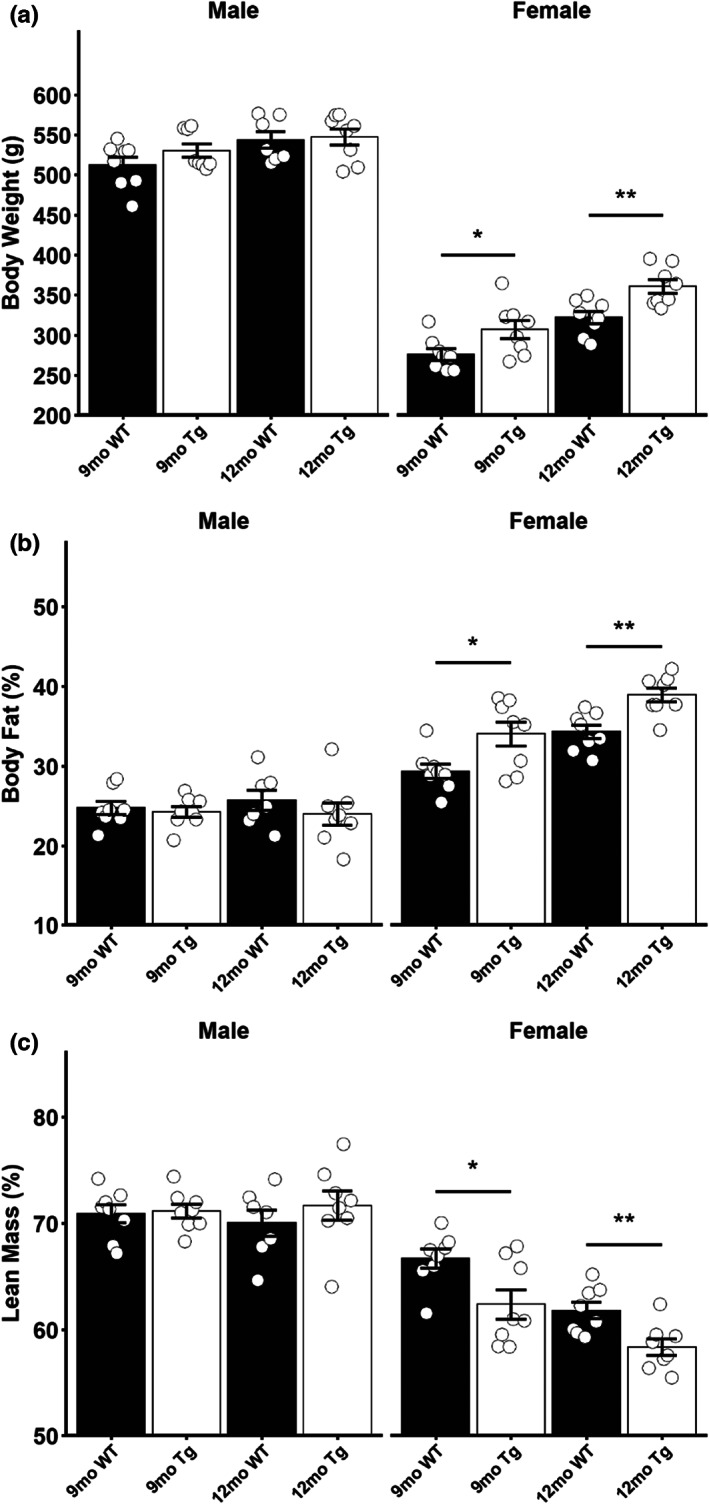
Altered body composition in female TgF344‐AD rats. (a) Body weight in male and female 9‐month‐old and 12‐month‐old rats. (b) Body fat percentage in male and female 9‐month‐old and 12‐month‐old rats. (c) Lean mass percentage in male and female 9‐month‐old and 12‐month‐old rats. Data presented as mean ± SEM with points representing individual rats. Statistical comparison was performed by two‐tailed *t*‐test with the welch correction applied where appropriate. WT‐wild‐type rats; Tg‐TgF344‐AD rats. *N* = 7–8 per group. *p < 0.05, **p < 0.01.

Quantitative magnetic resonance was used to assess body composition in males and females at both 9 months and 12 months of age. No significant differences in body weight, body fat percentage, or lean mass percentage were detected in 9‐ or 12‐month‐old male Tg rats compared to WT littermates (Figure 1a–c; Male). Interestingly, female Tg rats had a significantly greater body weight at both 9 (*t* = −2.3019, *p* = 0.0402) and 12 (*t* = −3.3787, *p* = 0.0046) months of age when compared to their WT littermates (Figure 1a; Female). This observed increase in body weight corresponds with disproportionate adipose accumulation, as the female Tg rats displayed elevated body fat percentages at both 9 (*t* = −2.6517, *p* = 0.0219) and 12 (*t* = −3.8238, *p* = 0.0019) months of age (Figure 1b; Female). The increased adiposity of the female Tg rats was accompanied by a reduction in the percentage of lean mass at both 9 (*t* = 2.6146, *p* = 0.0228) and 12 (*t* = 3.1701, *p* = 0.0068) months of age (Figure 1c; Female). Taken together, this suggests that sexual dimorphism exists in the body composition of the TgF344‐AD rat model with females accumulating more adipose tissue, at least at the ages examined.

**FIGURE 2:**
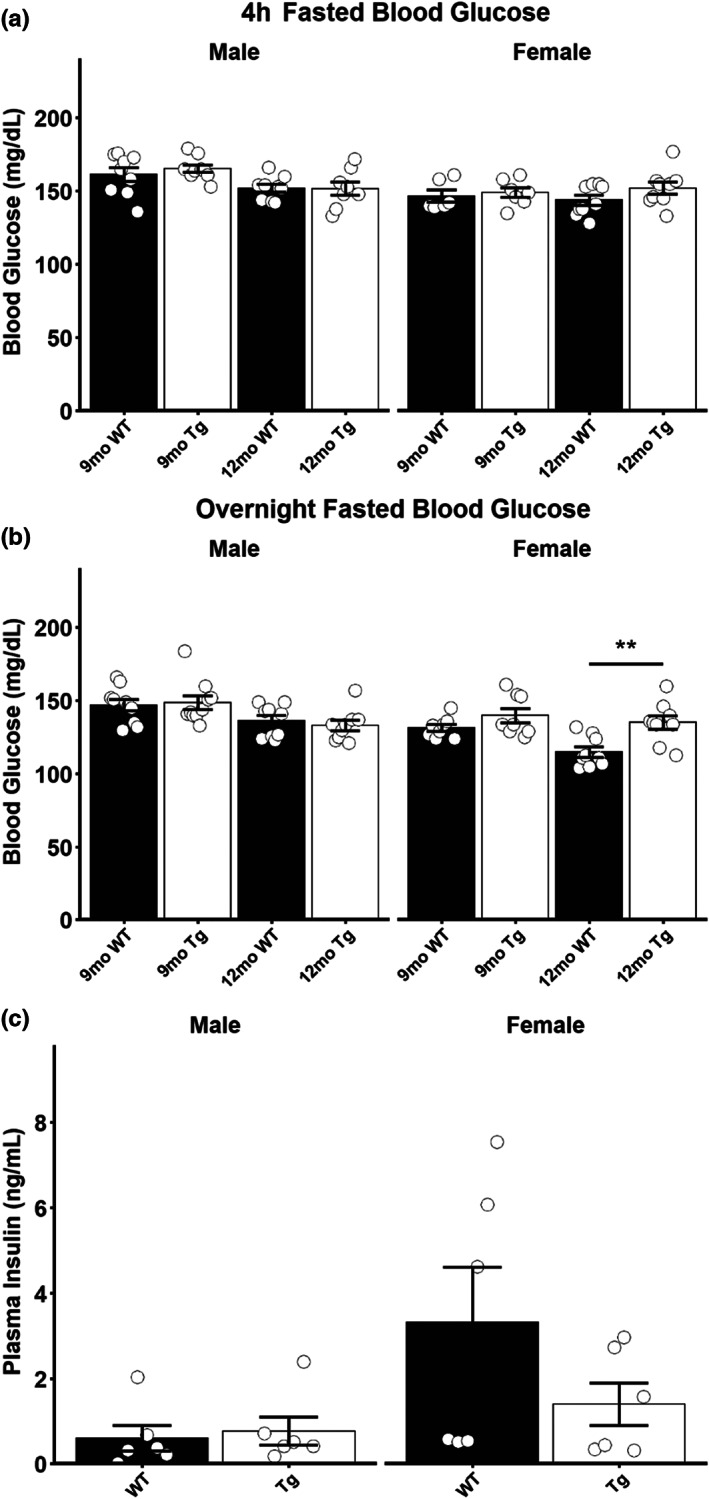
Elevated fasting glycemia in 12‐month‐old female TgF344‐AD rats. (a) Blood glucose in male and female 9‐month‐old and 12‐month‐old rats following a 4‐h fast. (b) Blood glucose in male and female 9‐month‐old and 12‐month‐old rats following an overnight fast. (c) Plasma insulin measurements in 12‐month‐old ad‐lib fed male and female rats. Data presented as mean ± SEM with points representing individual rats. Statistical comparison was performed by two‐tailed *t*‐test with the welch correction applied where appropriate. WT‐wild‐type rats; Tg‐TgF344‐AD rats. *N* = 6–9 per group for glycemia data, *N* = 6 per group for plasma insulin data. **p < 0.01.

To assess glycemic control in these rats, we investigated blood glucose levels following a short term (4 h) and an overnight fast. Following a short‐term fast, no differences in blood glucose were detected in any age or sex examined (Figure 2a). Following an overnight fast, no differences in blood glucose were seen in 9‐month‐old rats of either sex or 12‐month‐old male Tg rats (Figure 2b); however, 12‐month‐old female Tg rats displayed elevated blood glucose (*t* = −3.4784, *p* = 0.0034) following an overnight fast (Figure 2b; Female). This elevation in glycemia did not coincide with an increase in plasma insulin, as no differences in plasma insulin were detected between Tg and WT rats (Figure 2c).

**FIGURE 3:**
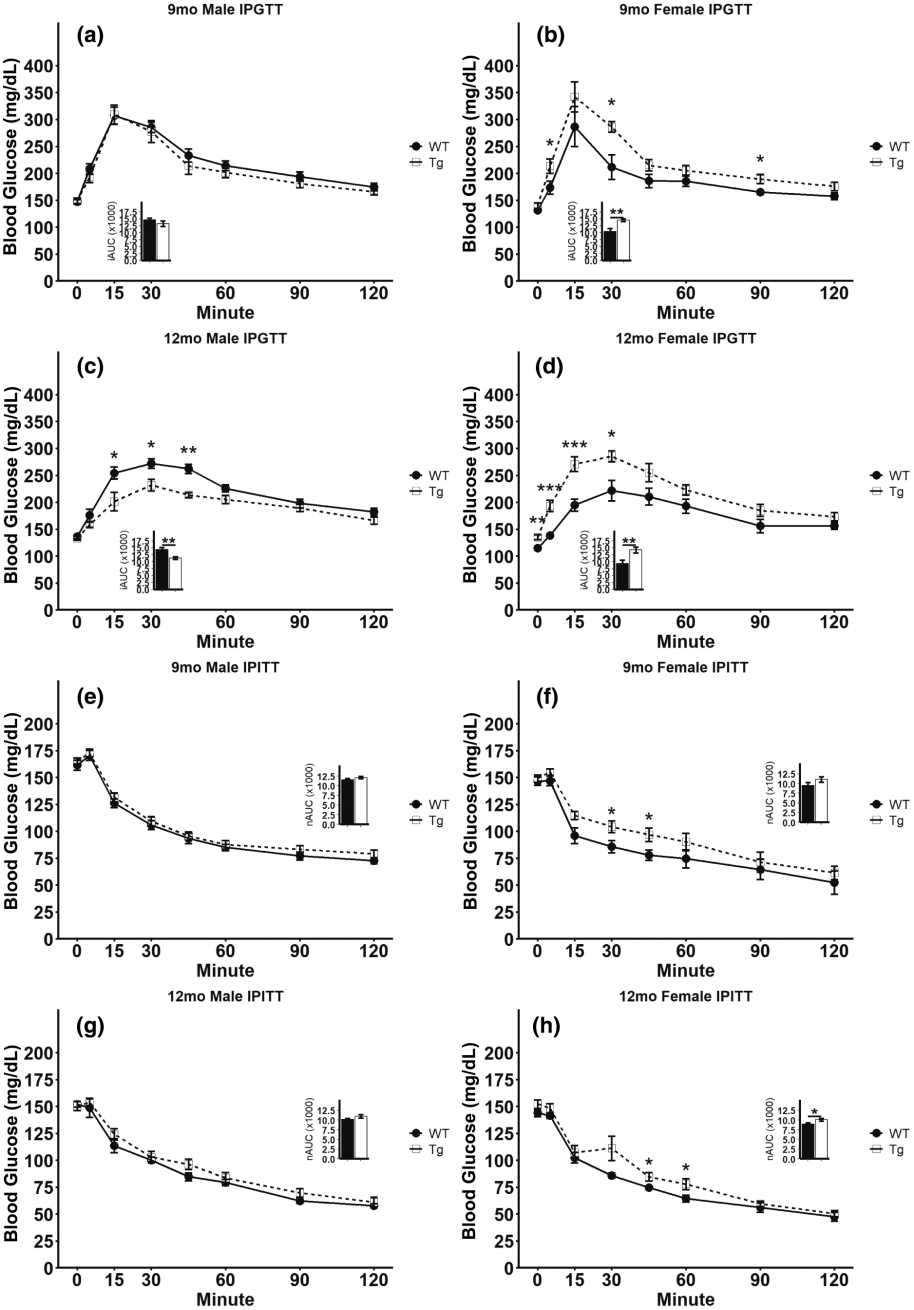
Disrupted glucose tolerance and insulin sensitivity in TgF344‐AD rats. (a–d) Glucose tolerance test in overnight fasted 9‐month‐old males (a) or females (b) and 12‐month‐old males (c) or females (d). Insets represent incremental area under the curve (iAUC) analyses for the data presented in (a–d). (e–h) Insulin tolerance test in 4 h fasted 9‐month‐old males (e) or females (f) and 12‐month‐old males (g) or females (h). Insets represent net area under the curve (nAUC) analyses for the data presented in e–h. Data presented as mean ± SEM with points representing individual rats. Statistical comparison was performed by two‐tailed *t*‐test with the welch correction applied where appropriate. WT‐wild‐type rats; Tg‐TgF344‐AD rats. *N* = 8–10 per group (a–d) or 6–9 per group (e–h). *p < 0.05, **p < 0.01, ***p < 0.001.

To directly investigate the glycemic response to a glucose or insulin bolus and more completely examine glucose homeostasis and insulin sensitivity in these rats, we carried out GTTs and ITTs following an overnight fast or 4‐h fast, respectively. GTTs in 9‐month‐old rats show no discernable differences between male Tg and WT rat glucose tolerance (Figure 3a), while female Tg rats displayed elevated blood glucose at 5, 30, and 90 min following an i.p. glucose challenge (Figure 3b), and significantly elevated (*t* = −3.4166, *p* = 0.0066) AUC at this age (Figure 3b inset). By 12 months of age, we observed improved glucose tolerance in male Tg rats with the Tg rats displaying lower blood glucose levels compared to WT littermates at 15, 30, and 45 min following a glucose challenge (Figure 3c) as well as a lower AUC (*p* = 0.0030; Figure 3c inset). In stark contrast to the males, 12‐month‐old female Tg rats continue to display impaired glucose tolerance, with significantly elevated glycemia at 5, 15, and 30 min following an i.p. glucose challenge (Figure 3d), and significantly elevated AUC (*t* = −3.0470, *p* = 0.0079) at this age (Figure 3d inset). ITTs revealed no difference in blood glucose following i.p. insulin challenge in male rats at either age examined (Figure 3e,g) and no difference in AUC (Figure 3e inset, 3g inset). Both 9‐month‐old and 12‐month‐old female Tg rats display reduced insulin sensitivity compared to WT littermates, with 9‐month‐old Tg rats displaying elevated blood glucose 30 and 45 min following insulin challenge and 12‐month‐old Tg rats displaying elevated blood glucose 45 and 60 min following insulin challenge (Figure 3f,h). 12‐month‐old female Tg rats also had greater AUC (*t* = −2.3483, *p* = 0.0324) compared to WT littermates (Figure 3h inset). Taken together, the GTT and ITT data suggest that sex‐specific differences in glucose tolerance and insulin sensitivity exist in the TgF344‐AD rat model, with Tg males displaying enhanced glucose tolerance and unaffected insulin sensitivity as age advances while Tg females display impairments in both parameters that further decline as age advances.

**FIGURE 4:**
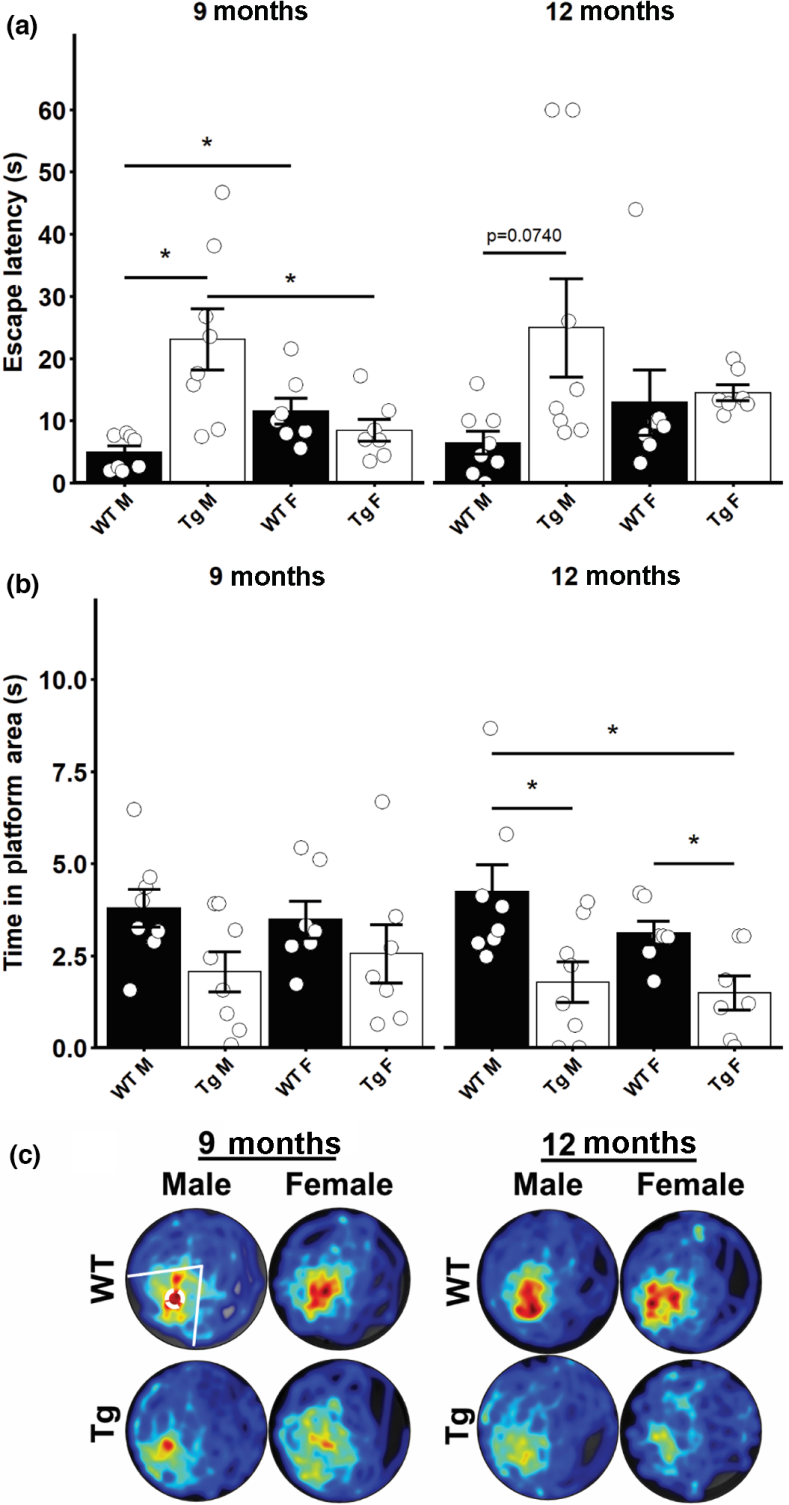
Reduced spatial learning and memory in TgF344‐AD rats. (a) Latency to escape on the 5th day of Morris Water Maze training in male and female 9‐month‐old and 12‐month‐old rats. (b) Duration of time spent in the area previously occupied by the platform following its removal during the probe trial of the Morris water maze in male and female 9‐month‐old and 12‐month‐old rats. (c) Merged heat maps of paths during the probe trial. Platform area and quadrant are highlighted in the upper left heatmap. Data presented as mean ± SEM with points representing individual rats. Statistical analysis was performed by two‐way ANOVA at each age. Post‐hoc analysis was carried out by pairwise comparisons with Benjamini and Hochberg correction applied. *p < 0.05 following adjustment for multiple comparison. *N* = 7–8 per group.

The Morris water maze was employed to assess spatial learning and long‐term memory. At 9 months of age, there was a significant main effect of genotype on the rat escape latency (*F* = 20.1691, *p* = 0.0001). In addition, a significant interaction of genotype and sex was detected at this age (*F* = 12.8381, *p* = 0.0014). Post‐hoc testing revealed Tg males had significantly greater escape latency than WT males and Tg females. In addition, WT females had significantly greater escape latency compared to WT males at this age (*p* < 0.05 all, Figure 4a; 9 months). In 12‐month‐old rats, a significant effect of genotype on the rat escape latency was detected (*F* = 7.2247, *p* = 0.0124). Post‐hoc testing revealed a tendency for 12‐month‐old Tg males to have a greater escape latency than their WT counterparts; however, this failed to reach significance after correction for multiple comparisons (Figure 4a; 12 months). During probe trials following the MWM training, a significant main effect of genotype on the time spent in the platform area was detected in the 9‐month‐old rats (*F* = 4.5486, *p* = 0.0426), but post‐hoc tests failed to detect significant differences between individual groups (Figure 4b,c; 9 months). In 12‐month‐old rats, a significant effect of genotype on the time spent in the platform area was detected (*F* = 10.4032, *p* = 0.0034). Post‐hoc testing revealed the Tg males and Tg females spent significantly less time in the platform area compared to WT littermates of the same sex, and that Tg females spent significantly less time in the platform area compared to WT males (Figure 4b,c; 12 months. *p* < 0.05 all). Taken together, these data suggest that male Tg rats have impaired learning evidenced by the elevated escape latency but intact spatial memory at 9 months old. By 12 months of age, male Tg rats display a trend for reduced learning, but significantly impaired spatial memory as indicated by poorer probe trial performance. 9‐month‐old female Tg rats display intact learning and memory, as no differences from WT rats were detected in either metric. At 12‐months old, the female Tg rat displayed no defect in learning, but significantly impaired spatial memory as their probe trial performance was worse compared to WT rats.

**FIGURE 5:**
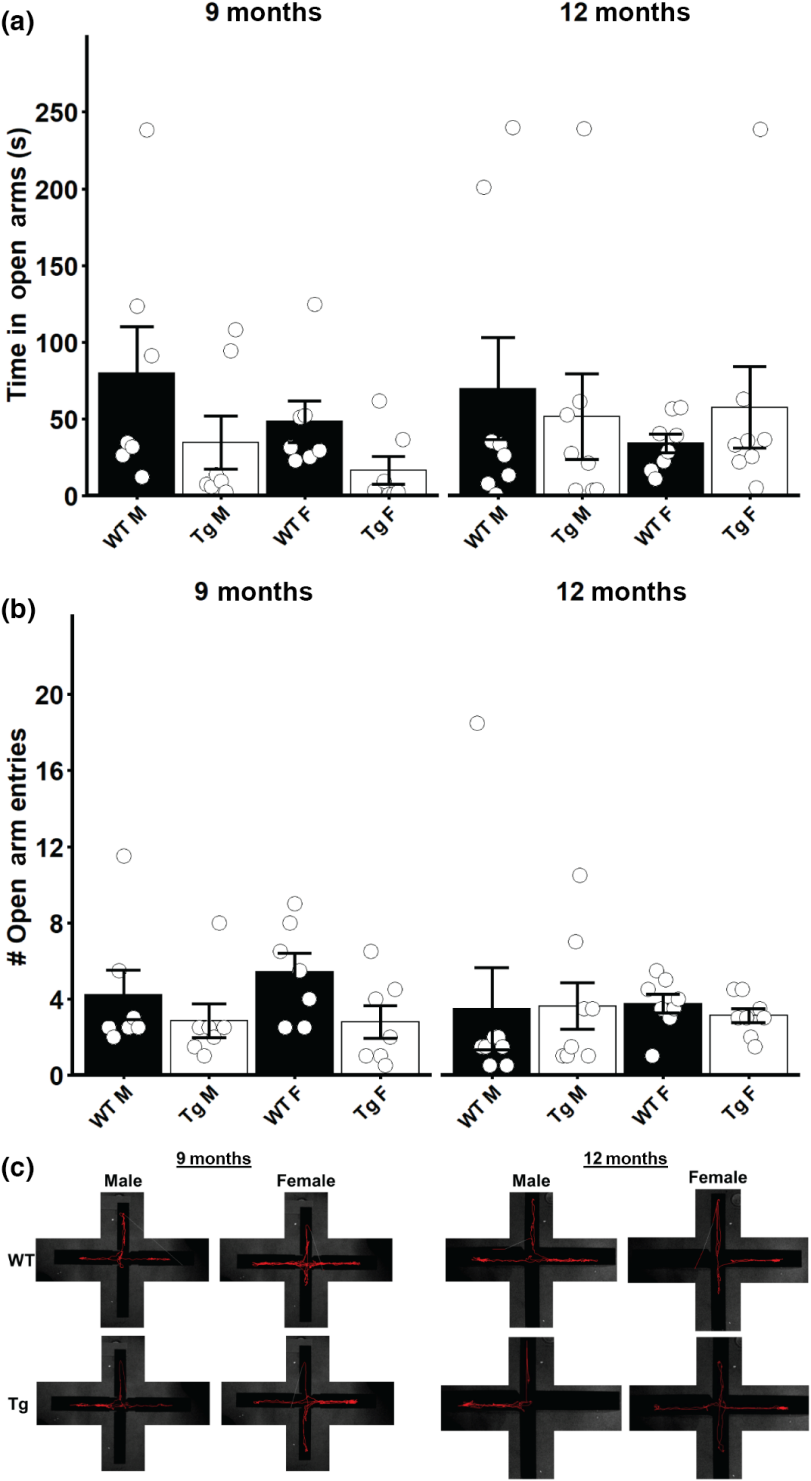
Elevated plus maze assessment of anxiety‐like behavior in TgF344‐AD rats. (a) Time spent in the open arms of the elevated plus maze for male and female 9‐month‐old and 12‐month‐old rats. (b) Number of entries into the open arms of the elevated plus maze for male and female 9‐month‐old and 12‐month‐old rats. (c) Representative traces of rat paths during the elevated plus maze. Vertical arms are the “open” arms; horizontal arms are the “closed” arms. Data presented as mean ± SEM with points representing individual rats. Statistical analysis was performed by two‐way ANOVA at each age. Post‐hoc analysis was carried out by pairwise comparisons with Benjamini and Hochberg correction applied. *N* = 7–8 per group.

**FIGURE 6:**
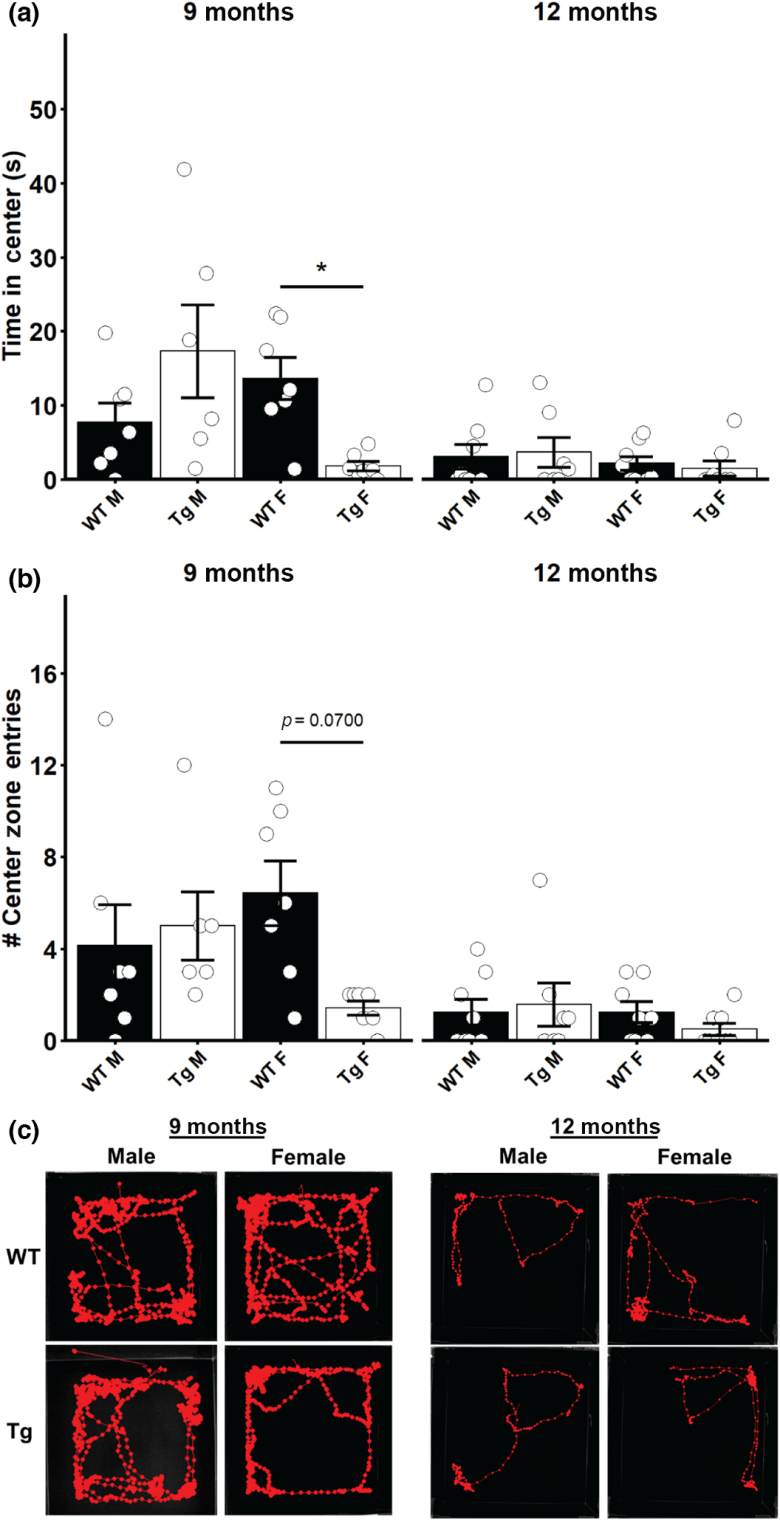
Open field test reveals anxiety‐like behavior in 9‐month‐old female TgF344‐AD rats. (a) Time spent in the center of the open field testing arena for male and female 9‐month‐old and 12‐month‐old rats. (b) Number of entries into the center zone of the open field testing arena for male and female 9‐month‐old and 12‐month‐old rats. (c) Representative traces of rat paths during the open field test. Data presented as mean ± SEM with points representing individual rats. Statistical analysis was performed by two‐way ANOVA at each age. Post‐hoc analysis was carried out by pairwise comparisons with Benjamini and Hochberg correction applied. *p < 0.05 following adjustment for multiple comparison. *N* = 6–8 per group.

We used EPM testing and OF testing to characterize the anxiety‐like behavior in this model. No differences were observed in the amount of time the 9‐month or 12‐month‐old male or female Tg rats spent in the open arms of the EPM compared to their WT littermates (Figure 5a). Similarly, we did not observe any differences in the number of open arm entries in any group examined (Figure 5b). When we carried out OF testing, we detected a significant interaction effect of genotype and sex on the cumulative time in the center of the OF in 9‐month‐old rats (*F* = 9.6933, *p* = 0.0049). Post‐hoc testing revealed that 9‐month‐old Tg female rats spent significantly less time in the center of the OF arena (*p* < 0.05, Figure 6a,c; 9 months). In 12‐month‐old rats, we did not detect any differences in the time spent in the center of the OF between any of the groups (Figure 6a,c; 12 months). When the frequency of entering the center zone of the OF was analyzed in 9‐month‐old rats, we detected a significant interaction of genotype and sex (*F* = 4.5982, *p* = 0.0428). Post‐hoc testing revealed a trend for Tg females to make fewer entries into the center of the OF testing arena, but this failed to reach significance (Figure 6b,c; 9 months). In 12‐month‐old rats, no difference was detected in the number of center zone entries for any group examined (Figure 6b,c; 12 months). Taken together, the EPM and OF tests suggest that younger TgF344‐AD female rats exhibit more anxiety‐like behaviors compared to their WT littermates, but as age advances, this difference disappears.

## 4 DISCUSSION

Identifying peripheral metabolic defects that precede or coincide with the onset of cognitive decline observed in AD represents an important avenue to fully understanding this disease. In this study, we report the first assessment of peripheral glucose tolerance and insulin sensitivity in conjunction with body composition and behavioral assessment in the TgF344‐AD rat model. Our results show sexual dimorphism in the body composition, glucose/insulin homeostasis, and behavioral phenotype for the assessments employed. Female Tg rats displayed adiposity, impaired glycemic control, and increased anxiety‐like behavior relative to their WT littermates before showing impairments in learning/memory. Male Tg rats conversely display body composition and insulin sensitivity that is comparable to their WT littermates while glucose tolerance was unchanged at 9 months of age and even improved by 12 months of age relative to WT controls. The male Tg rats also display impairments in learning/memory before these changes in peripheral metabolism and without displaying the anxious behavior seen in female Tg rats.

While reports on peripheral metabolic features in the TgF344‐AD rat are lacking, several accounts of perturbed glucose homeostasis exist in mouse models of AD. Transgenic *APP*‐expressing mouse models do not show alterations in glucose tolerance, and conflicting reports on insulin sensitivity exist with *hAPP23* transgenic males displaying impaired insulin tolerance at 4 months but not at 12 months (Hendrickx et al., 2021) while Tg2567 males and females display normal insulin sensitivity until 9 months old (Pedersen & Flynn, 2004). Male *APP/PS1* transgenic mice display reduced insulin sensitivity compared to littermates at 2 months of age (Macklin et al., 2017) and impaired glucose tolerance at 2, 5, 9, and 13 months but not at 15–18 months of age despite no significant changes in glycemic response to insulin being reported at these later time points (Denver et al., 2018; Kim et al., 2021; Macklin et al., 2017; Mody et al., 2011). When glucose homeostasis in *APP*, *PS1*, and *MAPT* triple transgenic 3xTg‐AD mice is examined, sex‐specific differences are seen in the glucose tolerance, with females reported to have impaired glucose tolerance at 6 months old while this was not seen in males (Robison et al., 2020). Studies that analyze sexes separately have reported impaired glucose tolerance in young 1‐ to 3‐month‐old males as well as 17‐ to 18‐month‐old males (Griffith et al., 2019) and 10‐ to 14‐month‐old females (Vandal et al., 2015); however, few studies have been carried out metabolic analyses in both males and females in the same analysis, making conclusions about differential effects of AD in males and females difficult to draw. Our data demonstrate that female TgF344‐AD rats display a phenotype associated with type 2 diabetes, with elevated adiposity and impaired glucose tolerance/insulin sensitivity. Males however do not display this phenotype and even display enhanced glucose tolerance in advanced age.

Defects in mitochondria structure and function have been reported in the TgF344‐AD rat model, with 18‐month‐old Tg animals displaying irregular mitochondrial morphology and increased mitochondrial fragmentation in preparations of hippocampus and brain cortex. These irregularities also correspond to lower SOD2 protein expression and elevated oxidative stress in these same regions (Yang et al., 2022). Furthermore, ex vivo assessment of hippocampal mitochondria activity has shown that both 6‐to‐7‐month‐old and 15‐to‐16‐month‐old Tg rats display reduced capacity for oxidative phosphorylation and decreased complex I activity compared to WT controls. The older group in this study also displayed reduced complex II activity relative to age‐matched WT controls (Viel et al., 2021), further demonstrating the presence of mitochondrial dysfunction in this model of AD. Taken together with our data, these demonstrate that altered metabolism, both central and peripheral, is a noteworthy feature in this model of AD. Further research is needed to demonstrate if these disruptions in metabolism directly influence behavioral deficits observed in this model.

Upon initial generation of the TgF344‐AD rat model, cognitive decline was reported in 15‐month‐old but not 6‐month‐old transgenic animals when the Barnes Maze was employed to assess learning and memory (Cohen et al., 2013). In another study where 13‐month‐old animals were assessed using the same test, no differences in maze escape latencies were detected between TgF344‐AD rats and their WT littermates but reversal trials, where the maze escape route is changed, revealed that the Tg rats took longer to escape the maze and used less effective search strategies (Morrone et al., 2020), indicating that as the task becomes more cognitively demanding deficits appear earlier in life. When Morris water maze testing, another assessment of learning and memory, is employed impaired cognition has been reported in Tg rats aged from 6 to 24 months old (Rorabaugh et al., 2017; Voorhees et al., 2018). An in‐depth analysis of Morris water maze performance revealed that Tg rats of both sexes had increased escape latencies and made more path deviations to the escape platform by 11 months old and that Tg rats generally perform wider, less direct movements during their swims (Berkowitz et al., 2018) leading the researchers to hypothesize that Tg rats become better at making wider, indirect movements during the Morris water maze. T‐maze testing for learning and memory indicates a similar timeline for cognitive decline, with 9‐ to 10‐month‐old Tg rats displaying cognitive decline (Tournier et al., 2021). Interestingly, a longitudinal analysis of T‐maze performance revealed no declines in cognition until 18 months of age in Tg animals and analyzing the sexes separately revealed that age had a significant effect in male but not female rats (Saré et al., 2020). Consistent with our data, we found that defects in cognition of male Tg rats occur by 9 months old while defects are not seen in females until 12 months of age and that these defects are more apparent in males. Cohen et al. (2013) initially reported that there were no differences in male or female Tg rats, and subsequent studies have either focused on a single sex or pooled sexes in their analysis. The cognitive differences between males and females reported here and by others highlight the importance of analyzing both sexes, as different assessments will likely uncover different aspects of the TgF344‐AD rat behavior and will provide important insight into the differential manifestation of AD in male and female patients.

We also observed sex‐specific differences in the anxiety‐like behavior in the Tg rats using the OF test and the EPM. Anxiety has been associated with amyloid‐β deposition before the cognitive decline in humans (Donovan et al., 2018), and elevated anxious behavior has been reported in the TgF344‐AD rat model upon initial generation (Cohen et al., 2013) and in subsequent studies at ages ranging from 4‐months to 12‐months old (Pentkowski et al., 2018, 2022; Saré et al., 2020; Wu et al., 2020), with Tg rats displaying less movement in the OF and fewer open arm entries of OF tests and EPM tests, respectively. Interestingly, our study only revealed differences in the 9‐month‐old female Tg rats while the other groups performed similarly to each other. It is possible that depression‐like behavior influenced our behavioral data, as depression has been reported in this rat model in both males and females (Tournier et al., 2021; Voorhees et al., 2018; Yang et al., 2022) when sucrose preference and forced swim tests are employed. No control for animal motivation was employed in our behavioral battery and thus differential effects of depression on the behavior of our groups cannot be ruled out as a potential confound and thus represents an important limitation of our study. As we and others have reported differential metabolic and behavioral features between the sexes of the TgF344‐AD rat, investigation into differential effects of depression in this model represents an important avenue for future research.

Of particular note in our study is the contrast between the peripheral glucose metabolism and behavioral assessment in males and females, with males displaying no impairments in glucose metabolism and even show enhanced glucose disposal at 12 months old while MWM indicated spatial learning and memory was impaired. Females, on the other hand, display impairments in peripheral glucose metabolism at both ages examined, but did not display deficits in spatial learning, and impairments in memory were not observed until 12 months. Similar discrepancies exist in examination of peripheral glucose metabolism in AD patients. Erythrocytes from individuals with AD have been shown to display elevated glycolytic activity compared to erythrocytes isolated from young and aged healthy controls (Kaminsky et al., 2013). Similarly, fibroblasts cultured from AD patients have shown increased capacity for glycolysis despite impairments in glucose uptake upon IGF‐I stimulation (Sonntag et al., 2017). More recently however, AD patient fibroblasts have shown reduced capacity for glycolysis (Bell et al., 2020). It is possible that differences in cell culture conditions could contribute to these discrepancies, as the fibroblasts displaying increased glycolytic capacity were grown glucose‐containing media (Sonntag et al., 2017) while the report of AD fibroblasts with reduced glycolytic capacity employed glucose‐free media (Bell et al., 2020), indicating that nutritional status may be an important driver for these changes. An additional explanation for these conflicts may be a combination of analyzing male and female data together along with differences in experimental group composition. The report of Bell and colleagues (2020) employed a control group with an equal number of males and females, while Sonntag et al. (2017) employed control groups with only a single female. These reports, in conjunction with the data presented here, indicate that perturbations in peripheral glucose metabolism do exist in AD; however, they are likely context dependent and may hinge on factors such as sex or diet. Indeed, further study is necessary to determine the mechanistic relationship between spatial learning/memory defects and impaired peripheral glucose metabolism, if one exists.

In conclusion, we report the first assessment of body composition, peripheral glucose tolerance, and peripheral insulin sensitivity in the TgF34‐AD rat model in conjunction with a behavioral battery to assess cognition and anxiety. While male Tg rats display normal body weight and glucose homeostasis, even showing improved glucose tolerance by 12 months old, female Tg rats display increased body weight due to increased adiposity and impaired glucose homeostasis at both ages tested. In contrast, male Tg rats display declines in cognition at 9 and 12 months old while females did not show defects until 12 months old. In addition, in younger female Tg rats, we found evidence for increased anxiety, while this was not detected in male rats. Taken together, our data suggest that a type 2 diabetes phenotype precedes the cognitive decline in females while males display cognitive decline before these differences in peripheral metabolism are detected in the TgF344‐AD model of Alzheimer's disease.

## AUTHOR CONTRIBUTIONS

Liou Y. Sun conceptualized the study, oversaw overall direction, and secured funding. Hemant Srivastava and Akash Nagarajan performed the experiments. A. Tate Lasher and Hemant Srivastava analyzed the data. A. Tate Lasher took the lead in writing the manuscript. A. Tate Lasher, Hemant Srivastava, and Liou Y. Sun edited the manuscript. All authors provided critical feedback and helped shape the research, analysis, and manuscript.

## FUNDING INFORMATION

This work was supported in part by the National Institute on Aging grants AG048264, AG057734, and AG050225 (L. Y. S.). The UAB Small Animal Phenotyping Core is supported by the NIH Nutrition & Obesity Research Center P30DK056336, Diabetes Research Center P30DK079626, and the UAB Nathan Shock Center P30AG050886A.

## CONFLICT OF INTEREST STATEMENT

All the contributing authors declared no conflicts of interest.

## Data Availability

The data supporting the findings of this study are available from the corresponding author upon reasonable request.
